# Pregnancy outcomes of diabetic women: Charting Oman’s progress towards the goals of the Saint Vincent Declaration

**DOI:** 10.4103/0256-4947.65253

**Published:** 2010

**Authors:** Mohammed N. Barakat, Randa M. Youssef, Jawad A. Al-Lawati

**Affiliations:** a Department of Noncommunicable Diseases Surveillance and Control, Ministry of Health, Oman; b Family Medicine and Public Health, Sultan Qaboos University, Oman

## Abstract

**BACKGROUND AND OBJECTIVES:**

Oman provides comprehensive care for the detection and management of diabetes during pregnancy with the goal of reducing or eliminating adverse outcomes for mothers and newborns. We assessed the outcome of pregnancies complicated with diabetes as compared to healthy controls.

**SUBJECTS AND METHODS:**

A 1-year retrospective review of registry records was conducted on pregnant women with gestational diabetes mellitus (GDM) and pre-gestational diabetes mellitus (PGDM). Of the 5394 women registered, 225 had GDM and 56 had PGDM. Fourteen cases of GDM and 2 cases of PGDM were excluded. For each patient recruited, the next healthy control of the same age and parity was selected.

**RESULTS:**

Nearly 80% of diabetic women achieved good glycemic control (hemoglobin A1c <7%). Adjusted for hypertension and body mass index, the risk of macrosomia was three times higher among women with GDM (OR=3.03, 95% CI=1.36-6.75) and up to seven times higher among those with PGDM (OR=7.20, 95% CI=2.30-22.61). A significantly higher risk of cesarean delivery was observed among women with GDM (OR=2.70, 95% CI=1.17-4.03) and PGDM (OR=4.39, 95% CI=1.68-11.49). Admission to the special care baby unit was higher among infants born to mothers with PGDM (OR=5.70, 95% CI=2.40-13.51) and GDM (OR=2.85, 95% CI=1.68-4.83).

**CONCLUSION:**

The findings indicate that many of the unfavorable pregnancy outcomes of diabetes for women and infants have not been brought under control despite the comprehensive care provided. Further studies are recommended to evaluate the system of care provided to pregnant women and to identify gaps in achieving the goals of the St. Vincent Declaration.

Diabetes is one of the most prevalent chronic diseases in Oman. The latest national figures revealed that the disease affects 12% of the population above the age of 20 years, with no preponderance with regard to either gender.^1^ At the time of pregnancy, nearly 3% of women develop gestational diabetes (GDM) (unpublished data; Ministry of Health, 2004).

Diabetes in pregnancy, either GDM or pre-gestational diabetes mellitus (PGDM), is linked to several maternal and fetal/neonatal complications. These include pregnancy-induced hypertension, preeclampsia, operative delivery, fetal macrosomia, neonatal asphyxia, birth trauma, respiratory distress, prematurity, and congenital defects.^2^

With the advance of medical care, higher rates of complications among diabetic women are no longer justified. In 1989, the Saint Vincent Declaration stated as a 5-year objective that the “outcome of diabetic pregnancy should approximate that of nondiabetic pregnancy”.^3^

The current study aims at assessing the outcome of pregnancies complicated with diabetes by comparing maternal and fetal outcomes in women with PGDM and GDM with those of healthy controls of nearly the same age and parity, in order to chart Oman’s progress in achieving the Saint Vincent Declaration target.

## SUBJECTS AND METHODS

A retrospective review of the delivery room records of Sohar Hospital was carried out for the year from 1 January to 31 December 2004 to extract from registries case details of women with GDM, PGDM (type 1 or type 2); healthy women served as the control group.

During the study period, of 5394 women in the labor room registry, 225 had GDM and 56 had PGDM. Women with PGDM were not clustered in the registry as having type 1 or type 2 diabetes; cross-checking of hospital records showed that 52 women had type 2 diabetes, and only 4 women had type 1 diabetes based on the 1999 WHO criteria.^4^ GDM was ascertained following the WHO screening protocol using the 75g, 2 hour oral glucose tolerance test (OGTT) during the first antenatal visit for women with a negative history of diabetes mellitus, which was repeated between weeks 24 and 28 of gestation for those with a negative OGTT.^4^ Of the identified diabetics, 12 women with GDM and 2 with PGDM type 1 were excluded because of incomplete information. Also, another 2 patients with GDM were excluded because of twin pregnancy.

All cases were reviewed for hypertension and classified as “pre-pregnancy hypertension” if the diagnosis had been established before the index pregnancy; and “pregnancy-induced hypertension” if blood pressure was ≥140/90 mm Hg following three consecutive readings after week 20 of gestation among previously normotensive subjects. Healthy controls with a singleton pregnancy and no evidence of hypertension or diabetes were identified from the same registry. For each diabetic women, the next healthy control of nearly the same age (±5 years) and parity (±2) was selected.

The management of gestational diabetes was by medical nutrition therapy and/or subcutaneous insulin. The target glycemic indices were 4-6 mmol/L before meals, 6-8 mmol/L 2 hours after meals (using capillary whole blood glucometers adjusted to give venous plasma readings) and glycosylated hemoglobin (A1c) less than 6.4% assessed by antibody immunoassay (reference interval, 4.1% to 6.4%). Ultimately, all women with GDM and PGDM enrolled in this study received insulin therapy.

Per hospital guidelines, labor was induced at 40 weeks of gestation if it was not spontaneous. Indications for cesarean delivery (CD) were failed induction, failed assisted delivery using ventouse or forceps, obstructed labor, breech presentation, fetal distress, large baby and a history of 2 or more CD deliveries. Indications for admission to the special care baby unit (SCBU) were prematurity (gestation age of <37 weeks), low Apgar score (<7 at 1 minute), low birth weight (<2.5 kg), presence of congenital anomalies, respiratory distress, birth asphyxia, and shoulder dystocia.

A transfer sheet was used to collect relevant data, including age, weight and height taken at the time of delivery; gravidity; parity; gestational age; and history of hypertension. This was in addition to outcome parameters, namely, mode of delivery; condition of perineum after labor; birth weight and health status of the newborn, including 1-minute Apgar score; clinically apparent congenital anomalies; and admission to SCBU. Data were analyzed using SPSS version 15. Mean, standard deviation and body mass index (BMI) were computed. Logistic regression analyses were used for quantification of associated risk by computing odds ratio (OR) and associated 95% confidence interval (95% CI) adjusted for BMI at full term and hypertension to eliminate their possible effects. The Chi-square and one-way analysis of variance were used to test significance of the obtained results at the 5% level.

## RESULTS

The distribution of the 512 women in relation to age, gravidity and parity is presented in Table 1. More than half of the women were between the age of 30 and 40 years. No significant differences were observed in the mean age of women in the three groups (F_2,509_ = 0.595, *P*=.552). Nearly equal proportions of women in the three groups reported gravidity and parity of more than six times (c24=2.557, *P*=.6345; and c24=5.962, *P*=.2020, respectively).

A statistically significant difference was observed between the three groups in BMI at full term (c24= 51.149, *P*=.0001). Less than half (41.22%) of the women in the control group had a BMI at full term equal to or greater than 30, as compared to nearly three quarters of the women with GDM (71.36%) and PGDM (75.93%). A statistically significant difference was observed (F_2,509_ =38.826, *P*=.0001) between women in the control group and those with GDM (*P*=.0001) and PGDM (*P*=.0001) but not between the two diabetic groups (*P*=.1884) (Table 1).

**Table 1:** Characteristics of pregnant women enrolled in the study.

Characteristic	Control (n= 245)		GDM (n= 213)		PGDM (n= 54)	
	No.	%	No.	%	No.	%
**Age in years**						
19 to <30	72	29.4	53	24.9	11	20.4
30 to <40	138	56.3	125	58.7	35	64.8
40 to 50	35	14.3	35	16.4	8	14.8

Mean (standard deviation)	32.8 (5.9)		33.4 (6.0)		33.5 (5.8)	
Range	20-46		20-50		19-45	

**Gravidity**						
1-3	43	17.6	38	17.8	8	14.8
4-6	50	20.4	49	23.0	8	14.8
≥7	152	62.0	126	59.2	38	70.4

Mean (standard deviation)	7.6 (3.7)		7.4 (3.6)		8.5 (3.8)	
Range	1-18		1-18		1-15	

**Parity**						
1-3	80	32.6	80	37.6	11	20.4
4-6	21	8.6	18	8.5	5	9.3
≥7	144	58.8	115	54.0	38	70.4

Mean (standard deviation)	6.2 (3.5)		5.9 (3.5)		7.0 (3.6)	
Range	0-17		0-14		0-14	

**Body mass index**						
< 25	51	20.8	19	8.9	4	7.4
25-	93	38.0	42	19.7	9	16.7
≥30	101	41.2	152	71.4	41	75.9

Mean (standard deviation)	29.3 (5.0)		33.2 (5.8)		34.8 (6.4)	
Range	19.2-45.4		19.3-48.8		22.6-53.5	

During the period of gestation, nearly 62% of women with GDM and PGDM had a mean A1c level of less than 6.4%, and nearly 18% had a mean A1c between 6.4% and 7% (but less than 7%). Less than a quarter (22.22%) of women with PGDM had pre-pregnancy hypertension as compared to only 4.69% of those with GDM (Table 2).

**Table 2:** Hemoglobin A1c and hypertension among women with gestational diabetes and pre-gestational diabetes.

	GDM (n= 213)		PGDM (n= 54)	
	No.	%	No.	%
**Hemoglobin A1c (%)**				
< 6.4	134	62.9	33	61.1
6.4 -	38	17.8	10	18.5
≥ 7	41	19.3	11	20.4

Mean (standard deviation)	6.3 (1.0)		6.3 (1.0)	
Range	3.5-11		4.8-9	

**Hypertension**					
Normotensive	151	70.9	27	50.0
Pregnancy-induced	52	24.4	15	27.8
Pre-pregnancy hypertension	10	4.7	12	22.2

Table 3 shows the risk of birth complications and outcome among women with GDM and PGDM as compared to the control group, adjusted for BMI and hypertension. Relative to the control group, women with GDM were nearly three times more likely to have a CD (OR=2.70, 95% CI=1.17-4.03). This risk was more than four times higher among those with PGDM (OR=4.39; 95% CI, 1.68-11.49). Excluding women delivered by CD, the risk of perineum episiotomy was insignificantly higher among women with GDM (OR=1.23; 95% CI, 0.39-3.93) and PGDM (OR=1.42; 95% CI, 0.17-12.15), while the risk of perineum tear was insignificantly lower among women with GDM (OR= 0.97; 95% CI, 0.54-1.76) and PGDM (OR=0.26; 95% CI, 0.03-2.02) as compared to the women in control group (Table 3).

**Table 3:** Estimated risk of birth complications and outcomes among enrolled women, adjusted for body mass index and hypertension.

Birth complication and outcome	Control (n= 245)		GDM (n= 213)		OR (95% CI)	PGDM (n= 54)		OR (95% CI)
	No.	%	No.	%		No.	%	
**Type of delivery**								
Spontaneous vaginal^a^	203	82.9	141	66.2	1.00	32	59.3	1.00
Assisted	21	8.6	16	7.5	0.52 (0.20-1.31)	2	3.7	0.51 (0.06-4.07)
Cesarean	21	8.6	56	26.3	2.70 (1.17-4.03)	20	37.0	4.39 (1.68, 11.49)
**Condition of perineum^b^**								
Intact^a^	174	77.7	118	75.2	1.00	31	91.2	1.00
Episiotomy	9	4.0	7	4.5	1.23 (0.39-3.93)	1	2.9	1.42 (0.17-12.15)
Tear	41	18.3	32	20.4	0.97 (0.54-1.76)	2	5.9	0.26 (0.03-2.02)
**Infant maturity**								
Full-term^a^	225	91.8	184	86.4	1.00	50	92.6	1.00
Preterm	19	7.8	29	13.6	0.92 (0.43-1.96)	4	7.4	0.77 (0.16-3.58)
Post-term	1	0.4	0	0.0		0	0.0	
**Birth weight**								
Normal^a^	219	89.4	161	75.6	1.00	33	61.1	1.00
Low birth weight	16	6.5	18	8.5	0.96 (0.39-2.37)	3	5.6	1.51 (0.32-7.20)
High birth weight	10	4.1	34	16.0	3.03 (1.36-6.75)	18	33.3	7.20 (2.30-22.61)
**Apgar score (1 minute)**								
≥ 7^a^	220	89.8	166	77.9	1.00	41	75.9	1.00
< 7	25	10.2	47	22.1	1.63 (0.88, 3.02)	13	24.1	1.89 (0.66-5.40)
**Congenital anomalies**								
Absent^a^	235	95.9	194	91.1	1	51	94.4	1
Present	10	4.1	19	8.9	1.62 (0.65-4.10)	3	5.6	1.51 (0.29-7.94)
**Infant outcome**								
Favorable^a^	238	97..1	198	93.0	1	52	96.3	1
Unfavorable	7	2.9	15	7.0	1.26 (0.41-3.91)	2	3.7	0.00 (0.0)
Still birth	6	2.5	3	1.4		1	1.6	
Birth asphyxia	0	0.0	3	1.4		0	0.0	
Respiratory distress	0	0.0	6	2.8		1	1.6	
Shoulder dystocia	1	0.4	3	1.4		0	0.0	
**Admission to SCBU**								
No^a^	214	87.4	141	66.2	1	27	50.0	1
Yes	31	12.7	72	33.9	2.85 (1.68-4.83)	27	50.0	5.70 (2.40-13.51)

a Reference category;

b Excluding cesarean delivery.

GDM and PGDM were not associated with a risk of preterm delivery. However, the estimated risk of giving birth to an infant with a high birth weight was three times higher among women with GDM (OR=3.03; 95% CI=1.36-6.75) and up to seven times higher among those with PGDM (OR=7.20; 95% CI=2.30-22.61) as compared to the women in control group (Table 3). Nearly a quarter (24.07%) of infants born to mothers with PGDM had an Apgar score of less than 7 at 1 minute. This proportion was slightly lower among infants born to mothers with GDM (22.07%) and much lower among infants born to healthy controls (10.20%). However, no excess risk was observed in this respect. Moreover, infants born to mothers with GDM and PGDM were not at higher risk of congenital anomalies or unfavorable outcomes; yet it is only among them that birth asphyxia and respiratory distress were encountered. These infants were at a significantly higher risk of being admitted to SCBU. This risk was much higher among infants born to mothers with PGDM (OR=5.70, 95% CI=2.40-13.51) as compared to those born to mothers with GDM (OR=2.85, 95% CI=1.68-4.83) (Table 3).

## DISCUSSION

In this series, women with GDM outnumbered those with PGDM. With the prevalent high parity (more than six, which inevitably means pregnancy at an older age) and the high BMI values based on readings at the time of delivery, which serve as a proxy for obesity as well as the coexistence of hypertension, these women and their unborn infants are at higher risk of adverse pregnancy outcomes unless efforts are taken to control their diabetes.

The ultimate goal of the specialized and comprehensive care provided to all pregnant women with diabetes is to maintain near-optimal blood glucose levels, which warrant a safe delivery. The mean value of glycosylated hemoglobin of 6.4% or even less reflects program success in empowering 80% of diabetic women in achieving blood sugar control and averting unfavorable pregnancy outcomes except for macrosomia, cesarean delivery and admission to SCBU.

Previous studies in Oman found an excess risk of macrosomia among older, obese, high-parity, euglycemic pregnant women as well as pregnant women who did not meet the criteria of GDM yet had a form of glucose intolerance (glucose challenge positive but OGTT negative).^5^,^6^ So in our cohort, the likelihood of macrosomia could be higher for both euglycemic and dysglycemic mothers. After controlling for the effects of age, parity, BMI and hypertension, an excess risk of fetal macrosomia, defined as a birth weight of more than 4 kg, has been observed among diabetic women. The rate of macrosomia was 16% among infants born to women with GDM, while it reached 33% among those born to women with PGDM. Other studies also reported higher rates of macrosomia and/or cases of “large for gestational age” among infants born to women with PGDM and GDM. 7–12 Rates of fetal macrosomia in association with diabetes show marked variation across studies because of the variation in the characteristics of the population studied, the extent of glycemic control and the adopted definition of macrosomia. In this study, the rate of macrosomia associated with GDM was found to be much lower than the 41% reported from the Netherlands, where fetal macrosomia was defined as birth weight above the 90th percentile; and the 28% reported among Asian Indian mothers, where a large baby was defined as one weighing >3.5 kg. However, the rates in our study were slightly higher than the 14% reported from Denmark by Jensen et al, who defined macrosomia as a birth weight of more than 4.5 kg.^9^,^13^,^14^ Also the current rate of macrosomia among Omani diabetic women is much higher than the 6.7% reported among the multi-ethnic population in the United Arab Emirates.^15^ Ethnicity by itself has its own effect on macrosomia.^16^ Macrosomia has been reported among 25% of Turkish women with mild pregnancy-induced carbohydrate intolerance managed only by diet modification, which is contrary to the situation in our cohort, where insulin was needed by all pregnant women.^17^

In our study, 26% of women with GDM and 37% of those with PGDM had a CD. This represented a threefold to fourfold increase when compared to controls. The rate of CD among women with GDM was higher than the 20% reported from the UAE and Saudi Arabia, but much lower than the figures reported from Iran, Pakistan and Netherlands, where the rates ranged from 43% to 75%.^10^–^13^,^15^,^18^ Among women with PGDM, the rate of CD was lower than the 45% reported from the UAE.^8^ Combining the rates of CD among women with GDM and PGDM, the overall figure is lower than the 48% reported from Saudi Arabia or the recorded fivefold increase when compared to nondiabetics.^19^,^20^

In maternal diabetes, macrosomia is the main reason for CD. Mathew et al found that macrosomia doubled the risk of CD.^5^ Studies that reported higher rates of macrosomia also reported higher rates of CD.^10^,^13^ A review of the literature concluded that among diabetic women, the intent of CD is to avoid complications.^21^ This is very much true as diabetic women in this study who had a vaginal delivery had a lower tendency toward perineum tear.

The incidence of preterm labor in association with diabetes varies from less than 10% among Asians to 30% to 40% in Caucasians.^9^,^12^,^22^,^23^ In our cohort, 86.38% of women with GDM and 92.59% of women with PGDM were able to carry their fetuses to full term. Newborns of diabetic women were significantly more likely to be admitted to SCBU for specialized care. Nearly a quarter of these infants had an Apgar score of less than 7 at 1 minute, which justified admission especially if a lower Apgar score was recorded thereafter. Furthermore, it is only among this group that birth asphyxia and respiratory distress were reported, in addition to the relatively higher rates of birth defects.

This study relied on a retrospective review of records of delivery room and linkage to records of diabetic clinic to extract relevant information. This approach limited our knowledge of the condition of newborn in terms of Apgar score after 1 minute and status at discharge to determine final outcome. Findings indicate that many of the unfavorable pregnancy outcomes of diabetes in women and infants have not been brought under control despite the comprehensive care provided. Further studies are recommended to evaluate the system of care provided to pregnant women and to identify gaps that should be bridged to achieve the goals of Saint Vincent Declaration.
